# The Effect of Inflammation on the Formation of Thyroid Nodules

**DOI:** 10.1155/2020/9827349

**Published:** 2020-07-10

**Authors:** Zexin Li, Yiteng Huang, Xiang Chen, Chiju Wei, Peixuan Yang, Wencan Xu

**Affiliations:** ^1^Health Care Center, The First Affiliated Hospital of Shantou University Medical College, Shantou 515041, China; ^2^Multidisciplinary Research Center, Shantou University, Shantou 515041, China; ^3^Department of Endocrinology, The First Affiliated Hospital of Shantou University Medical College, Shantou 515041, China

## Abstract

**Background:**

Some studies have demonstrated that inflammation is highly associated with the prevalence of thyroid nodules (TNs). However, more confounders, such as metabolic diseases, should be adjusted.

**Methods:**

A clinical study collecting 2722 subjects was conducted to confirm the association between inflammation and TNs. The underlying mechanism was investigated in combination with bioinformatics analysis.

**Results:**

In the clinical study, propensity score matching was used to match metabolic parameters and other confounders, and it is observed that subjects with high inflammation had a higher prevalence of TNs and thyroid-stimulating hormone (TSH) than those with low inflammation. After further matching TNs, it is found that inflammation was positively associated with TSH, which was also demonstrated in a population without TNs. In bioinformatics study, inflammation did not promote TNs formation directly. Instead, it inhibited the synthesis of thyroid hormone, which might be the cause of the elevated TSH coexisting with inflammation.

**Conclusion:**

Inflammation promotes the development of TNs disease, probably due to its indirect effect through inhibiting the synthesis of thyroid hormone, which results in the elevation of TSH.

## 1. Introduction

Thyroid nodules (TNs) disease, an increasing diagnosis in clinical practice during the past decades, is a thyroid disorder more common than Graves' disease and thyroiditis in global population. The prevalence of TNs in adults is found from 3% to 7% with neck palpation. Nevertheless, with more sensitive examination means such as ultrasound, the prevalence of TNs ranges from 13% to 67% in subjects who undergo a routine health checkup [1]. This figure even reaches up to 68% when the frequency of the ultrasonic probe is higher (13 MHz) [2]. Most of the TNs are asymptomatic and benign, but a possibility of potential malignancy cannot be excluded. Approximately 5%–13% of patients with TNs are at high risk of malignancy when they are detected by ultrasound, CT, or MRI [3]. Thus, it is necessary to find out the risk factors of TNs and avoid them.

The causes of TNs are various, from iodine intake, radiation exposure, and tobacco smoking to metabolic status [4–7]. Inflammation can impair thyroid tissue and cause thyroiditis directly [8]. In contrast, inflammation also can promote tissues and cells hyperplasia. Cytokines, active molecules in chronic inflammation, play critical roles in widely regulating cellular functions and promoting cellular proliferation, differentiation, and survival [9]. In recent decades, some studies have found a close relationship between inflammation and TNs. A study including 988 euthyroid subjects from China showed that *Helicobacter pylori* (HP) infection significantly increases the risk of TNs, probably due to the elevated inflammation levels *in vivo* resulted from HP infection [10]. Another study found that patients with chronic infection of hepatitis C virus (HCV) have a higher prevalence of papillary thyroid cancer, the malignancy of TNs, than healthy people. The underlying mechanism may be due to the upregulating expression and secretion of *CXCL10* in thyroid cells, which recruit T-helper one that secretes interferon-*γ* (IFN-*γ*) and tumour necrosis factor-*α* (TNF-*α*), both of which create an inflammatory circumstance in the thyroid gland [11]. An association between TNs and inflammation can be found not only in patients with chronic infection but also in people without apparent infection. A study has found that children and adolescents with nontoxic nodular goitre have a slightly increased concentration of cytokines (interleukin-6 and interleukin-8) in serum without elevated antithyroid antibodies [12]. Furthermore, inflammation is also associated with the malignant growth in TNs [13]. All of these studies suggest that inflammation may facilitate the prevalence of TNs. However, all of these studies did not take the confounders metabolic parameters into account. Metabolic diseases are highly prevalent in recent decades. People with metabolic disorders, such as metabolic syndrome (MS), were reported to have a higher prevalence of TNs [14]. For this reason, we designed a clinical study to investigate the association between TNs and inflammation, and in combination with a bioinformatics study, we further explored the underlying mechanism.

## 2. Methods

### 2.1. Clinical Study

#### 2.1.1. Study Design and Subjects

A clinical study was designed to confirm the association between TNs and inflammation. We retrospectively and randomly collected 2722 subjects who underwent health checkups in our department in the First Affiliated Hospital of Shantou University Medical College from December 2014 to November 2018. The subjects were from the local population, living in a coastal, universal iodised salt city, and were considered as iodine adequate. All of the subjects underwent thyroid ultrasound, thyroid function, and other physical and laboratory examinations. The fasting blood samples were obtained between 7:30 AM and 9:30 AM. Based on thyroid ultrasound and thyroid function examination, individuals who had a thyroid surgery history, thyroiditis, Graves' disease, or thyroid dysfunction were excluded. Meanwhile, individuals with severe diseases and infection were excluded. Thyroid dysfunction was defined as serum thyroid-stimulating hormone (TSH) >5.5 mIU/L or <0.35 mIU/L, free thyroxine (free T4, FT4) >22.7 pmol/L or <11.5 pmol/L, and/or free triiodothyronine (free T3, FT3) >6.5 pmol/L or <3.5 pmol/L. After exclusion, a total of 2068 eligible individuals were enrolled in this study. The collected information included age, gender, creatinine (Cr), metabolic and inflammatory parameters, thyroid function, and presence or absence of TNs. TN disease was defined as any nodular lesion that is distinct from the normal parenchyma of the thyroid gland by ultrasound in this context [15]. Metabolic parameters comprised body mass index (BMI), systolic blood pressure (SBP), diastolic blood pressure (DBP), fasting blood sugar (FBS), uric acid (UA), triglyceride (TG), cholesterol (CHOL), high-density lipoprotein (HDL), and low-density lipoprotein (LDL). Inflammatory parameters comprised white blood cell (WBC), neutrophil (NE), lymphocyte (LY), monocyte (Mo), NE/LY ratio, LY/Mo ratio, and Mo/HDL ratio. These traditional inflammatory markers, which are inexpensive and extensively used in clinical practice, can reflect the systemic inflammation. In case of infection, most of these parameters increase significantly. In some noninfectious diseases with low-grade inflammation, some of these parameters will increase at some level. Patients with diabetes, who are considered to have low-grade inflammation *in vivo*, show increased WBC, NE, and LY [16]. Patients with Graves' disease, a chronic autoimmune disease, have higher LY and Mo in blood [17]. Thus, WBC, NE, LY, and Mo can suggest the systemic inflammation in subjects without obvious infection. In this study, we mainly used these four parameters to assess the inflammatory levels. All procedures performed in studies involving human participants were in accordance with the ethical standards of the institutional and/or national research committee and with the 1964 Helsinki Declaration and its later amendments or comparable ethical standards. The study was approved by the Ethics Committee of the First Affiliated Hospital of Shantou University Medical College.

### 2.2. Bioinformatics Study

#### 2.2.1. Microarray Data Searching and Downloading

To explore the possible mechanism how inflammation was involved in the formation of TNs, we searched datasets on National Centre for Biotechnology Information Gene Expression Omnibus (GEO) database (https://www.ncbi.nlm.nih.gov/gds/) by using the keywords “thyroid” and “inflammation” or “cytokine.” Eventually, datasets GSE6339 and GSE5054 were obtained. Dataset GSE6339 was presented by Jean-Fred Fontaine. It contained 183 samples from 2 cell lines and 12 types of human thyroid tissue, including samples from the normal thyroid, oncocytic carcinoma, follicular carcinoma, papillary carcinoma, atypical follicular adenoma, atypical oncocytic adenoma, macrofollicular adenoma, microfollicular adenoma, oncocytic adenoma, multinodular goitre, Graves' disease, and autoimmune thyroiditis. Dataset GSE5054 was presented by Rork Kuick. It came from a conditioned experiment, in which normal primary thyroid cells were incubated separately with the vehicle, 100 IU/ml interferon-*γ* (IFN-*γ*), 50 IU/ml interleukin-1*β* (IL-1*β*), and a combination of IFN-*γ* and IL-1*β* for 24 or 72 hours. The experiment was repeated five times using thyroid cells from five different patients and contained 20 samples in total.

#### 2.2.2. Microarray Data Preprocessing

The series matrix files of the datasets were downloaded. Dataset GSE6339 had been processed by print-tip Lowess normalisation and median normalisation. Dataset GSE5054 had been processed by the quantile-normalised trimmed-mean method and log-transformed with log(max(*x*+50,0)+50) using base 10 logarithms. Dataset GSE6339 was used to analyse gene expression differences between TNs (a combination of thyroid carcinoma, thyroid adenoma, and multinodular goitre) and normal thyroid tissue. The cell line, Graves' disease, and autoimmune thyroiditis samples were excluded, and a total of 159 samples were retained for analysis. After excluding the outlier samples and filtering genes with too many missing samples or zero variance, 156 samples and 6464 genes were chosen for weighted gene coexpression network analysis (WGCNA), a network-based gene screening method that has been widely used to determine the candidate biomarkers or therapeutic targets [18].

#### 2.2.3. Identification of the Soft-Thresholding Power and Construction of Weighted Gene Coexpression Network

The soft-thresholding power was determined to satisfy the scale-free network. We used the pickSoftThreshold function of WGCNA package to calculate the scale-free topology fit index and mean connectivity of each power. The optimal power value (*β* = 4) was picked for gene coexpression network construction using the blockwiseModules function in WGCNA package. The parameters of blockwiseModules function were set as follows:power = 4, maxBlockSize = 6464, minModuleSize = 30, TOMType = “unsigned,” reassignThreshold = 0, mergeCutHeight = 0.25, corType = “Pearson,” and verbose = 3.

#### 2.2.4. Identification of Clinical Significant Modules and Hub Genes

Pearson's correlation was used to analyse the association between modules and clinical traits of TNs. Clinical traits, including age, gender, and nodule type, were extracted from dataset 6339. Nodule type included non-TNs, singular TNs, and multiple TNs according to the development of TNs. The significant modules were identified by the correlation between nodule type and eigengenes (MEs), which represented all genes in a given module. The most correlated module with nodule type was considered as the most significant and meaningful module, which was worth further analysis. Hub genes in the clinically significant module were identified by a combination of WGCNA and protein-protein interaction (PPI) network. In WGCNA, hub genes had high within-module connectivity (cor.geneModuleMembership > 0.8) and high relation with nodule type (cor.geneTraitSignificance > 0.2), both of which were measured by Pearson's correlation and presented by an absolute value. In the PPI network, hub genes were considered as the top ten genes calculated by maximal clique centrality and a degree algorithm in cytoHubba software. The finally determined hub genes were considered as the intersection of hub genes identified by WGCNA and the PPI network.

#### 2.2.5. Association between Hub Genes and Inflammation

Dataset GSE5054, concerning gene expression alteration of thyroid cells under stimulation of inflammatory factors, was used to detect the associations between hub genes and inflammation.

### 2.3. Statistical Analysis

SPSS 19.0 statistical software (SPSS Inc., Chicago, IL, USA) and R software (v3.5.3) were used for statistical analysis. Continuous data were given as mean (SD), and dichotomous data were given as number (%). Q-Q plot was used to detect whether the continuous data were normally distributed. For better comparisons of clinical characteristics, propensity score matching (PSM) with nearest-neighbour matching was used to account for the baseline differences. Standardised mean difference (SMD) was calculated to observe the differences between groups and examine whether the PSM reduced the differences before and after matching. Data between groups were compared by the *t*-test, Wilcoxon test, or *χ*^2^ test according to the type and distribution of the data. Stepwise logistic regression (direction = “both”) was used to detect the independent impact factors of TNs. The mRNA expression levels of genes between groups were compared by the *t*-test, analysis of variance, Wilcoxon test, or Kruskal–Wallis test according to the group count and data distribution. Differences with *P* values less than 0.05 were considered as statistically significant.

## 3. Results

### 3.1. Clinical Study

#### 3.1.1. Subject Characteristics

A total of 2068 subjects were enrolled in this study and divided into two groups: TNs (986 subjects) and non-TNs (1082 subjects). The baseline of the clinical characteristics of the subjects is illustrated in Table 1. Compared to the subjects without TNs, the subjects with TNs had higher age, female proportion, BMI, SBP, DBP, FBS, TG, CHOL, WBC, LY, and Mo, but lower Cr, UA, TSH, FT3, and FT4.

#### 3.1.2. Association between TNs, Inflammation, and Thyroid Function

Step logistic regression was carried out to find some inflammatory parameters, which were related to TNs, and the result showed that blood LY was most associated with TNs among the inflammatory parameters (Supplementary Table S1). According to the inflammatory levels, the subjects were divided into two groups, high-LY and low-LY groups, using the median of LY count as a cutoff. The subjects with high LY had higher inflammatory levels, such as higher WBC, NE, LY, Mo, LY/Mo, and Mo/HDL, and had higher Cr, BMI, SBP, DBP, FBS, UA, TG, CHOL, LDL, TSH, and FT3, but lower female proportion, HDL, and NE/LY. In addition, as we expected, a higher prevalence of TNs was observed in the high-LY group than in the low-LY group (51.7% vs. 43.6%, *P* < 0.001; Table 2). Based on PSM, which was used to account for the confounders age, gender, Cr, and metabolic parameters such as BMI, SBP, DBP, FBS, UA, TG, CHOL, HDL, and LDL, a higher prevalence of TNs was still observed in the high-LY group than in the low-LY group (52.3% vs. 43.9%, *P*=0.001; Table 3). In Table 3, higher TSH and FT3 are detected in the high-LY group as well. It was summarised that inflammation had a positive relation with the prevalence of TNs, TSH, and FT3.

To investigate the association between inflammation and thyroid function, we used PSM to match the potential confounders, such as age, gender, Cr, metabolic parameters, and TNs. Then we found that, in the overall population (including TNs and non-TNs), the subjects in the high-LY group had higher TSH and FT3 (Table 4). This result was a little different from that in the non-TNs population. In the non-TNs population, after matching age, gender, Cr, and metabolic parameters, TSH and FT4 were higher in the high-LY group than in the low-LY group (Supplementary Table S2). Based on the results of the overall and non-TNs population, it was concluded that inflammation was positively associated with TSH.

### 3.2. Bioinformatics Study

#### 3.2.1. Weighted Gene Coexpression Network Construction and Key Module and Hub Genes Identification

Although clinical study indicated that inflammation might be involved in the prevalence of TNs, due to the elevated TSH coexisting with inflammation, more evidence was needed to demonstrate the effect of inflammation on TNs. To detect whether inflammation could promote the formation of TNs directly, first, we found out the hub genes of TNs, the alteration of which highly correlate with TNs. Then, we tried to figure out whether the hub genes had identical alteration under the stimulation of inflammatory factors in an experiment *in vitro*. To seek the hub genes that were involved in the formation and development of TNs, we performed WGCNA by R software. One hundred and fifty-six samples including normal thyroid tissues and thyroid nodule tissues (thyroid carcinoma, thyroid adenoma, and multinodular goitre) in dataset GSE6339 were clustered by the hierarchical cluster analysis using the hclust function (method = “average”) (Supplementary Figure S1). To construct a scale-free coexpression network, we chose a power of *β* = 4 (scale-free *R*^2^ near 0.85) as the soft-threshold (Supplementary Figure S2). A total of 25 modules, ranging from 37 to 1811 in sizes, were identified by the hierarchical clustering tree analysis. Grey60 module was found to have the top correlation with the nodule type (Supplementary Figure S3). This module was considered to have the highest association with the formation and development of TNs. Three hub genes HBB, HBA1, and HERC3 in grey60 module were identified by a combination of WGCNA and the PPI network, and they had a strong positive correlation between each other and a tendency to coexpress (Supplementary Figure S4). Thus, hub genes HBB, HBA1, and HERC3 were considered as the biomarkers of TNs.

#### 3.2.2. Association between Hub Genes and Inflammation

**Figure 1:**
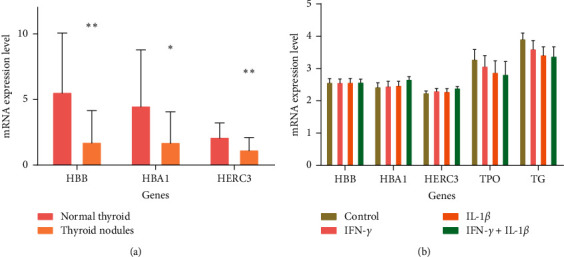
Association between hub genes and inflammation. (a) mRNA expression levels of hub genes in TNs. Hub genes in TNs had lower expression than normal thyroid tissue. (b) Expression of hub genes, TPO, and TG in thyroid cells stimulated by cytokines. The stimulation of cytokines could downregulate the expression of genes TPO and TG rather than hub genes. ^*∗*^*P* < 0.05; ^*∗∗*^*P* < 0.001.

Dataset GSE5054 was further analysed to detect the association between hub genes and inflammation. In dataset GSE6339, compared to the normal thyroid tissue, mRNA expression levels of hub genes were all downregulated in TNs (Figure 1(a)), which implicated that the downregulation of the hub genes had an important role in the development of TNs. However, in dataset GSE5054, the mRNA expression levels of hub genes did not show significant alteration under the stimulation of cytokines (Figure 1(b)), which suggested that cytokines could not promote the development of TNs directly. Instead, genes TG and TPO, both of which were key genes of thyroid hormone synthesis, were downregulated although statistically insignificant (Figure 1(b)). In the bioinformatics study, although a direct effect of inflammation on TNs formation was not found, an inhibiting effect on the synthesis of the thyroid hormone was observed.

## 4. Discussion

Although several previous studies have suggested a link between inflammation and TNs, all of these studies did not take metabolic diseases into account [10–12]. Metabolic syndrome (MS) is characterised by a cluster of metabolic diseases. The relationship between MS and TNs is emerging and brought into focus in recent years. A study evaluated the association between TNs and MS and its components, such as obesity and hyperglycemia, in apparently healthy Koreans and found that the presence of MS or some of its components is closely associated with the occurrence of TNs. A positive linear correlation is detected between the relevant number of MS components and the presence of TNs [14]. It is reported that patients with MS living in an iodine-deficient environment have significantly increased prevalence of TNs and thyroid volume. Insulin resistance, important pathogenesis of MS, contributes to this increased risk substantially, and it is an independent risk factor of TNs [19]. Therefore, a correction of metabolic diseases in this study is necessary.

In this study, we found that subjects in the high-LY group had higher WBC, NE, LY, and Mo, which implied that these subjects had higher inflammation in the body. They also had a higher prevalence of TNs following the correction of metabolic parameters, which showed a close association between inflammation and TNs. Meanwhile, we found that subjects in the high-LY group had higher TSH. This result implied that the development of TNs might be due to the elevated TSH, which has been demonstrated to promote the growth and proliferation of thyroid cell in different ways [20–22], leading to the goitre and TNs. After further correcting TNs, which might have an effect on thyroid function, by matching TNs or choosing the non-TNs, a higher TSH was still observed in the subjects with high LY. Thus, it is inferred that TNs are probably caused by inflammation, which coexists with the elevated TSH.

In our knowledge, there have been no studies focusing on how inflammation induces TNs disease. Although our clinical study implies a possible mechanism concerning the elevated TSH, it is still unknown whether inflammation can stimulate TNs formation directly. Thus, we were referred to the public databases GEO. Dataset GSE6339 was searched and downloaded from the GEO database. WGCNA was used to screen out the significant gene module associated with the development of TNs. The PPI network combined with WGCNA was used to identify the hub genes of TNs. Eventually, HBB, HBA1, and HERC3 were identified as the hub genes, and they were downregulated in TNs.

HBB and HBA1 have been demonstrated to be downregulated in several cancers, which is related to cancer cell growth. In ovarian cancer, HBB and HBA1 are downregulated by at least ten folds when compared to normal ovarian tissues [23]. In anaplastic thyroid cancer cell lines (ACL), HBB expression is also found significantly decreased. When it is upregulated by transfection, cell growth is significantly suppressed [24]. The decrease of ubiquitin ligase HERC3 can increase NFKB nuclear import and NFKB-dependent transcription, which are critical for cell growth and proliferation [25]. Hence, the downregulation of HBB, HBA1, and HERC3 are highly related to thyroid cell growth and TNs formation. Unfortunately, dataset GSE5054 from an experiment *in vitro* did not show obvious downregulation of hub genes in thyroid cells under stimulation with cytokines, which implied that inflammation, at least the cytokines (IFN-*γ* and IL-1*β*) used in the experiment, do not cause TNs directly. Fortunately, in this dataset, a decreased trend of the expression of TG and TPO, both of which are key genes for the synthesis of thyroid hormone, was found. As we have known, the lower thyroid hormone will lead to TSH synthesis and secretion [26]. Thus, the link between inflammation and the elevated TSH is probably the reduction of thyroid hormone induced by inflammation. This finding will support the clinical study.

There is a complicated interaction between inflammation and thyroid function. Inflammation can cause thyroid dysfunction, and thyroid hormones regulate inflammatory response [27]. During severe illness including infection characterised with significantly elevated inflammation, serum thyroid hormones decrease with or without the decrease of TSH, known as euthyroid sick syndrome, not only in humans but also in other animals such as mice, rats, and rabbits [28]. The lower T3 can be interpreted by the alteration of iodothyronine deiodinases, while the lower T4 is due to the inhibition of TSH [29]. However, in the case of normal TSH, it needs a better explanation for the lower T4. The explanation can be found in the bioinformatics study. The lower T4 can be caused by inflammation directly. However, the feedback regulation of pituitary-thyroid axis, the increased TSH induced by the decreased T4, does not work in these severe diseases. However, it works in subclinical, chronic, and low-grade inflammation. Moura Neto et al. [30] compared the thyroid dysfunction between type 1 (T1DM, an autoimmune disease) and type2 diabetes (T2DM, a metabolic disease with low-grade inflammation [31]) and found that both serum T3 and T4 decrease in both diseases, and TSH decreases in T1DM but increases in T2DM.

## 5. Conclusion

Inflammation is a risk factor of TNs, probably due to its indirect effect through inhibiting the synthesis of thyroid hormone, which results in the elevation of TSH. For the prevention of the formation and development of TNs, which probably lead to a surgery or a thyroid dysfunction, some anti-inflammatory measures may be applicable for patients suffering from low-grade inflammation.

## Figures and Tables

**Table 1:** Clinical characteristics of all subjects with and without TNs.

Variables	TNs (*n* = 986)	Non-TNs (*n* = 1082)	*P*	SMD
Age (years)	47.23 (13.99)	36.93 (11.32)	<0.001^*∗∗*^	0.809
Gender (female, *n*)	467 (47.4)	254 (23.5)	<0.001^*∗∗*^	0.516
Creatinine (mmol/L)	75.10 (22.58)	77.08 (17.38)	<0.001^*∗∗*^	0.098
Body mass index (kg/m^2^)	24.04 (6.55)	23.52 (2.84)	0.002^*∗*^	0.104
Systolic blood pressure (mmHg)	122.87 (13.86)	118.53 (12.16)	<0.001^*∗∗*^	0.333
Diastolic blood pressure (mmHg)	79.45 (9.57)	77.75 (9.94)	<0.001^*∗∗*^	0.174
Fasting blood sugar (mmol/L)	5.56 (1.09)	5.26 (1.02)	<0.001^*∗∗*^	0.277
Uric acid (mmol/L)	392.35 (98.97)	401.44 (97.05)	0.035^*∗*^	0.093
Triglyceride (mmol/L)	1.38 (0.84)	1.34 (1.05)	<0.001^*∗∗*^	0.036
Cholesterol (mmol/L)	5.03 (0.89)	4.78 (0.88)	<0.001^*∗∗*^	0.281
High-density lipoprotein (mmol/L)	1.42 (0.33)	1.39 (0.33)	0.113	0.070
Low-density lipoprotein (mmol/L)	2.99 (0.76)	2.79 (0.74)	<0.001^*∗∗*^	0.263
Thyroid stimulating hormone (mIU/L)	1.79 (0.96)	1.90 (0.90)	0.005^*∗*^	0.124
Free triiodothyronine (nmol/L)	5.15 (0.58)	5.20 (0.53)	0.031^*∗*^	0.095
Free thyroxine (nmol/L)	15.76 (2.18)	16.23 (2.20)	<0.001^*∗∗*^	0.215
White blood cell (10^9^ cells/L)	6.55 (1.72)	6.34 (1.56)	0.003^*∗*^	0.131
Neutrophil (10^9^ cells/L)	3.64 (1.25)	3.54 (1.19)	0.064	0.081
Lymphocyte (10^9^ cells/L)	2.32 (0.67)	2.23 (0.58)	0.001^*∗*^	0.148
Monocyte (10^9^ cells/L)	0.40 (0.13)	0.39 (0.12)	0.041^*∗*^	0.090
Neutrophil/lymphocyte ratio	1.66 (0.73)	1.67 (0.64)	0.767	0.005
Lymphocyte/monocyte ratio	6.22 (2.27)	6.10 (1.92)	0.514	0.057
Monocyte/high-density lipoprotein ratio	0.30 (0.14)	0.30 (0.13)	0.516	0.029

Data were presented as mean (SD) or number (%). TNs: thyroid nodules; SMD: standardized mean difference. ^*∗*^*P* < 0.05; ^*∗∗*^*P* < 0.001.

**Table 2:** Clinical characteristics of subjects with high and low lymphocyte.

Variables	High-LY (*n* = 1034)	Low-LY (*n* = 1034)	*P*	SMD
Age (years)	42.12 (13.96)	41.56 (13.36)	0.353	0.041
Gender (female, *n*)	331 (32.0)	390 (37.7)	0.007^*∗*^	0.120
Creatinine (mmol/L)	78.05 (17.66)	74.23 (22.03)	<0.001^*∗∗*^	0.191
Body mass index (kg/m^2^)	24.00 (2.93)	23.53 (6.38)	0.033^*∗*^	0.094
Systolic blood pressure (mmHg)	122.22 (13.67)	118.99 (12.45)	<0.001^*∗∗*^	0.247
Diastolic blood pressure (mmHg)	79.73 (9.89)	77.39 (9.58)	<0.001^*∗∗*^	0.241
Fasting blood sugar (mmol/L)	5.45 (1.14)	5.35 (0.97)	0.032^*∗*^	0.095
Uric acid (mmol/L)	415.02 (95.07)	379.20 (97.76)	<0.001^*∗∗*^	0.371
Triglyceride (mmol/L)	1.53 (1.10)	1.19 (0.75)	<0.001^*∗∗*^	0.356
Cholesterol (mmol/L)	5.05 (0.89)	4.75 (0.87)	<0.001^*∗∗*^	0.337
High-density lipoprotein (mmol/L)	1.37 (0.31)	1.44 (0.35)	<0.001^*∗∗*^	0.224
Low-density lipoprotein (mmol/L)	3.00 (0.74)	2.78 (0.76)	<0.001^*∗∗*^	0.289
Thyroid stimulating hormone (mIU/L)	1.91 (0.96)	1.79 (0.90)	0.006^*∗*^	0.120
Free triiodothyronine (nmol/L)	5.24 (0.53)	5.12 (0.57)	<0.001^*∗∗*^	0.213
Free thyroxine (nmol/L)	16.10 (2.19)	15.91 (2.22)	0.057	0.084
White blood cell (10^9^ cells/L)	7.25 (1.60)	5.64 (1.24)	<0.001^*∗∗*^	1.127
Neutrophil (10^9^ cells/L)	3.85 (1.28)	3.33 (1.10)	<0.001^*∗∗*^	0.434
Lymphocyte (10^9^ cells/L)	2.75 (0.50)	1.79 (0.28)	<0.001^*∗∗*^	2.374
Monocyte (10^9^ cells/L)	0.43 (0.13)	0.35 (0.11)	<0.001^*∗∗*^	0.667
Neutrophil/lymphocyte ratio	1.42 (0.47)	1.91 (0.77)	<0.001^*∗∗*^	0.762
Lymphocyte/monocyte ratio	6.76 (1.97)	5.55 (2.04)	<0.001^*∗∗*^	0.601
Monocyte/high-density lipoprotein ratio	0.34 (0.14)	0.26 (0.12)	<0.001^*∗∗*^	0.576
Thyroid nodules (*n*)	535 (51.7)	451 (43.6)	<0.001^*∗∗*^	0.163

Data were presented as mean (SD) or number (%). LY: lymphocyte; SMD: standardized mean difference. ^*∗*^*P* < 0.05; ^*∗∗*^*P* < 0.001.

**Table 3:** Association between inflammation and TNs based on propensity sore matching.

Variables	High-LY (*n* = 803)	Low-LY (*n* = 803)	*P*	SMD
Age (years)	41.86 (14.13)	42.02 (13.35)	0.814	0.012
Gender (female, *n*)	267 (34.1)	269 (34.4)	0.958	0.005
Creatinine (mmol/L)	76.48 (17.26)	76.58 (22.88)	0.458	0.005
Body mass index (kg/m^2^)	23.72 (3.00)	23.64 (2.61)	0.58	0.028
Systolic blood pressure (mmHg)	120.37 (11.99)	120.60 (12.13)	0.707	0.019
Diastolic blood pressure (mmHg)	78.40 (8.94)	78.53 (9.27)	0.777	0.014
Fasting blood sugar (mmol/L)	5.41 (1.12)	5.43 (0.99)	0.940	0.024
Uric acid (mmol/L)	396.70 (87.31)	395.78 (94.51)	0.84	0.010
Triglyceride (mmol/L)	1.31 (0.67)	1.27 (0.71)	0.066	0.046
Cholesterol (mmol/L)	4.87 (0.79)	4.89 (0.86)	0.679	0.021
High-density lipoprotein (mmol/L)	1.40 (0.30)	1.41 (0.33)	0.518	0.033
Low-density lipoprotein (mmol/L)	2.88 (0.68)	2.90 (0.75)	0.636	0.024
Thyroid stimulating hormone (mIU/L)	1.90 (0.98)	1.80 (0.88)	0.048^*∗*^	0.100
Free triiodothyronine (nmol/L)	5.24 (0.54)	5.15 (0.56)	0.002^*∗*^	0.156
Free thyroxine (nmol/L)	16.09 (2.23)	15.96 (2.24)	0.247	0.059
White blood cell (10^9^ cells/L)	7.14 (1.54)	5.69 (1.26)	<0.001^*∗∗*^	1.032
Neutrophil (10^9^ cells/L)	3.78 (1.22)	3.36 (1.11)	<0.001^*∗∗*^	0.357
Lymphocyte (10^9^ cells/L)	2.72 (0.48)	1.80 (0.28)	<0.001^*∗∗*^	2.344
Monocyte (10^9^ cells/L)	0.43 (0.12)	0.36 (0.11)	<0.001^*∗∗*^	0.612
Neutrophil/lymphocyte ratio	1.41 (0.47)	1.91 (0.75)	<0.001^*∗∗*^	0.799
Lymphocyte/monocyte ratio	6.74 (1.92)	5.52 (1.84)	<0.001^*∗∗*^	0.648
Monocyte/high-density lipoprotein ratio	0.32 (0.13)	0.27 (0.12)	<0.001^*∗∗*^	0.431
Thyroid nodules (*n*)	409 (52.3)	343 (43.9)	0.001^*∗*^	0.170

Matching variables included age, gender, body mass index, systolic blood pressure, diastolic blood pressure, fasting blood sugar, uric acid, creatinine, triglyceride, cholesterol, high-density lipoprotein, and low-density lipoprotein. Data were presented as mean (SD) or number (%). LY: lymphocyte; SMD: standardized mean difference. ^*∗*^*P* < 0.05; ^*∗∗*^*P* < 0.001.

**Table 4:** Association between inflammation and thyroid function based on propensity sore matching.

Variables	High-LY (*n* = 770)	Low-LY (*n* = 770)	*P*	SMD
Age (years)	42.13 (14.21)	41.90 (13.59)	0.746	0.017
Gender (female, *n*)	261 (33.9)	264 (34.3)	0.914	0.008
Creatinine (mmol/L)	76.07 (17.32)	76.32 (23.22)	0.539	0.012
Body mass index (kg/m^2^)	23.66 (2.87)	23.62 (2.65)	0.793	0.013
Systolic blood pressure (mmHg)	120.64 (12.95)	120.51 (12.07)	0.829	0.011
Diastolic blood pressure (mmHg)	78.64 (9.45)	78.37 (9.39)	0.574	0.029
Fasting blood sugar (mmol/L)	5.43 (1.23)	5.40 (0.94)	0.450	0.023
Uric acid (mmol/L)	396.74 (83.16)	395.46 (96.51)	0.78	0.014
Triglyceride (mmol/L)	1.33 (0.81)	1.30 (0.82)	0.099	0.042
Cholesterol (mmol/L)	4.89 (0.83)	4.88 (0.87)	0.851	0.010
High-density lipoprotein (mmol/L)	1.41 (0.31)	1.40 (0.33)	0.797	0.013
Low-density lipoprotein (mmol/L)	2.89 (0.70)	2.90 (0.74)	0.74	0.017
Thyroid stimulating hormone (mIU/L)	1.90 (0.97)	1.77 (0.89)	0.007^*∗*^	0.138
Free triiodothyronine (nmol/L)	5.24 (0.53)	5.14 (0.57)	0.001^*∗*^	0.177
Free thyroxine (nmol/L)	16.12 (2.17)	16.00 (2.23)	0.266	0.057
White blood cell (10^9^ cells/L)	7.15 (1.52)	5.67 (1.25)	<0.001^*∗∗*^	1.067
Neutrophil (10^9^ cells/L)	3.80 (1.25)	3.35 (1.11)	<0.001^*∗∗*^	0.378
Lymphocyte (10^9^ cells/L)	2.71 (0.45)	1.80 (0.28)	<0.001^*∗∗*^	2.424
Monocyte (10^9^ cells/L)	0.43 (0.13)	0.36 (0.11)	<0.001^*∗∗*^	0.625
Neutrophil/lymphocyte ratio	1.43 (0.49)	1.92 (0.77)	<0.001^*∗∗*^	0.754
Lymphocyte/monocyte ratio	6.73 (2.01)	5.48 (2.03)	<0.001^*∗∗*^	0.615
Monocyte/high-density lipoprotein ratio	0.32 (0.13)	0.27 (0.12)	<0.001^*∗∗*^	0.421
Thyroid nodules (*n*)	364 (47.3)	373 (48.4)	0.683	0.023

Matching variables included age, gender, body mass index, systolic blood pressure, diastolic blood pressure, fasting blood sugar, uric acid, creatinine, triglyceride, cholesterol, high-density lipoprotein, low-density lipoprotein, and thyroid nodules. Data were presented as mean (SD) or number (%). LY: lymphocyte; SMD: standardized mean difference. ^*∗*^*P* < 0.05; ^*∗∗*^*P* < 0.001.

## Data Availability

The datasets generated during and/or analysed during the current study are available from the corresponding author upon reasonable request.

## Additional Points

*Limitation*. In the clinical study, we have included some confounders, such as age, gender, renal function, and metabolic parameters, all of which were demonstrated to be associated with TNs [7, 14, 32]. However, confounders, such as radiation exposure and smoke, were not taken into account, and this may have an impact on the results. Further study is needed to exclude the impact of these confounders. In bioinformatics analysis, data in datasets GSE5054 were log-transformed using base 10 logarithms, which would largely reduce the differences and result in statistical insignificance when comparing expression levels of TG and TPO between groups. Nonetheless, we still accepted the trend the data showed.

## Supplementary Materials

Supplementary Materials Supplementary Table S1: independent impact factors for thyroid nodules in the euthyroid population. Supplementary Table S2: association between inflammation and thyroid function in subjects without TNs based on propensity sore matching. Supplementary Figure S1: clustering dendrogram of 156 samples. Supplementary Figure S2: determination of soft-thresholding power (*β*) in WGCNA. Supplementary Figure S3: identification of key modules associated with nodule type of TNs. Supplementary Figure S4: correlations between hub genes.
