# Supplementary material for: The Effect of Inflammation on the Formation of Thyroid Nodules
Source: Int J Endocrinol. 2020 Jul 10;2020:9827349. doi: 10.1155/2020/9827349 (PMC7368952; doi:10.1155/2020/9827349)

Article title The effect of inflammation on the formation of thyroid nodules

Journal name Journal of cancer research and clinical oncology

Author names Zexin Li, Yiteng Huang, Xiang Chen, Chiju Wei, Peixuan Yang, Wencan Xu

Corresponding author Wencan Xu, Department of Endocrinology, the First Affiliated Hospital of Shantou University Medical College; e-mail: xuwcan@163.com

Supplementary Table S1. Independent impact factors for thyroid nodules in the euthyroid population

**Variables**	**β**	**SE**	**Wald χ^2**	***P***	**OR**	**95% CI of OR**
Age	0.063	0.005	13.675	<0.001**	1.065	[1.055, 1.075]
Gender	1.517	0.135	11.253	<0.001**	4.560	[3.509, 5.953]
Systolic blood pressure	0.025	0.007	3.616	<0.001**	1.025	[1.011, 1.039]
Diastolic blood pressure	-0.024	0.009	-2.834	0.005*	0.976	[0.960, 0.992]
Uric acid	0.001	0.001	1.999	0.046*	1.001	[1.000, 1.002]
Cholesteral	-0.278	0.128	-2.166	0.030*	0.757	[0.587, 0.972]
Thyroid stimulating hormone	-0.206	0.055	-3.731	<0.001**	0.814	[0.730, 0.907]
Free triiodothyronine	0.309	0.101	3.075	0.002*	1.362	[1.119, 1.661]
Lymphocyte	0.281	0.092	3.064	0.002*	1.324	[1.107, 1.587]

β: partial regression coefficient; SE: standard error of partial regression coefficient; OR: odds ratio; CI: confidence interval. Stepwise logistic regression was performed. Dependent variable was thyroid nodules and independent variables were age, gender, body mass index, systolic blood pressure, diastolic blood pressure, fasting blood sugar, uric acid, creatinine, triglyceride, cholesterol, high-density lipoprotein, low-density lipoprotein, thyroid-stimulating hormone, free triiodothyronine, free thyroxine, neutrophil, lymphocyte, and monocyte. *$P < 0.05$; **$P < 0.001$.

Supplementary Table S2. Association between inflammation and thyroid function in subjects without TNs based on propensity sore matching

**Variables**	**High-LY (*n* = 407)**	**Low-LY (*n* = 407)**	***P***	**SMD**
Age (years)	36.74 (11.87)	36.84 (10.95)	0.905	0.012
Gender (female, *n*)	93 (22.9)	90 (22.1)	0.867	0.018
Creatinine (mmol/L)	77.42 (17.99)	77.02 (16.79)	0.739	0.005
Body mass index (kg/m^2)	23.59 (2.80)	23.56 (2.84)	0.862	0.028
Systolic blood pressure (mmHg)	118.82 (12.18)	119.05 (10.78)	0.783	0.019
Diastolic blood pressure (mmHg)	77.95 (10.18)	78.14 (8.80)	0.774	0.014
Fasting blood sugar (mmol/L)	5.31 (1.25)	5.27 (0.94)	0.298	0.024
Uric acid (mmol/L)	405.01 (89.87)	402.30 (94.01)	0.674	0.010
Triglyceride (mmol/L)	1.28 (0.73)	1.27 (0.74)	0.497	0.046
Cholesterol (mmol/L)	4.78 (0.77)	4.79 (0.88)	0.798	0.021
High-density lipoprotein (mmol/L)	1.39 (0.31)	1.39 (0.34)	0.806	0.033
Low-density lipoprotein (mmol/L)	2.83 (0.67)	2.83 (0.77)	0.947	0.024
Thyroid stimulating hormone (mIU/L)	1.95 (0.92)	1.82 (0.85)	0.037*	0.100
Free triiodothyronine (nmol/L)	5.24 (0.52)	5.17 (0.54)	0.056	0.156
Free thyroxine (nmol/L)	16.44 (2.20)	16.08 (2.14)	0.019*	0.059
White blood cell (10^9 cells/L)	7.11 (1.53)	5.71 (1.25)	<0.001**	1.032
Neutrophil (10^9 cells/L)	3.78 (1.29)	3.38 (1.12)	<0.001**	0.357
Lymphocyte (10^9 cells/L)	2.68 (0.41)	1.81 (0.27)	<0.001**	2.344
Monocyte (10^9 cells/L)	0.43 (0.12)	0.36 (0.11)	<0.001**	0.612
Neutrophil/lymphocyte ratio	1.43 (0.51)	1.91 (0.69)	<0.001**	0.799
Lymphocyte/monocyte ratio	6.70 (1.98)	5.41 (1.64)	<0.001**	0.648
Monocyte/high-density lipoprotein ratio	0.33 (0.14)	0.28 (0.12)	<0.001**	0.431

Matching variables included age, gender, body mass index, systolic blood pressure, diastolic blood pressure, fasting blood sugar, uric acid, creatinine, triglyceride, cholesterol, high-density lipoprotein, and low-density lipoprotein. Data were presented as mean (S.D.) or number (%). TNs: thyroid nodules; LY: lymphocyte; SMD: standardised mean difference. *P < 0.05; **P < 0.001.

Supplementary Fig S1 Clustering dendrogram of 156 samples

One hundred and fifty-six samples were clustered using average linkage and Pearson's correlation methods, and their clinical traits were shown as a heatmap. No obvious outlier samples were found in these samples.

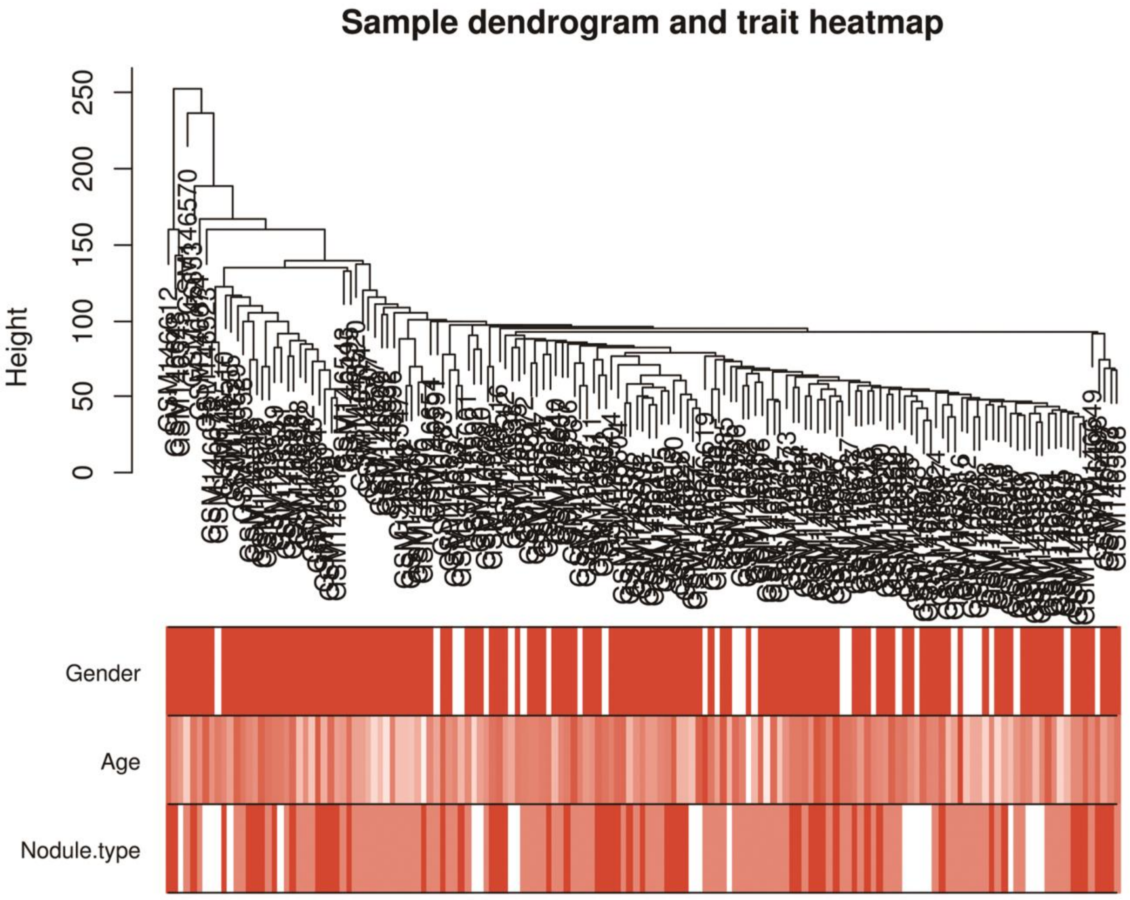

Supplementary Fig S2 Determination of soft-thresholding power (β) in WGCNA

(a) Analysis of the scale-free fit index for different β values. β values over the red line (R^2 = 0.85) were suitable for a scale-free topology model. (b) Analysis of the mean connectivity of different β values.

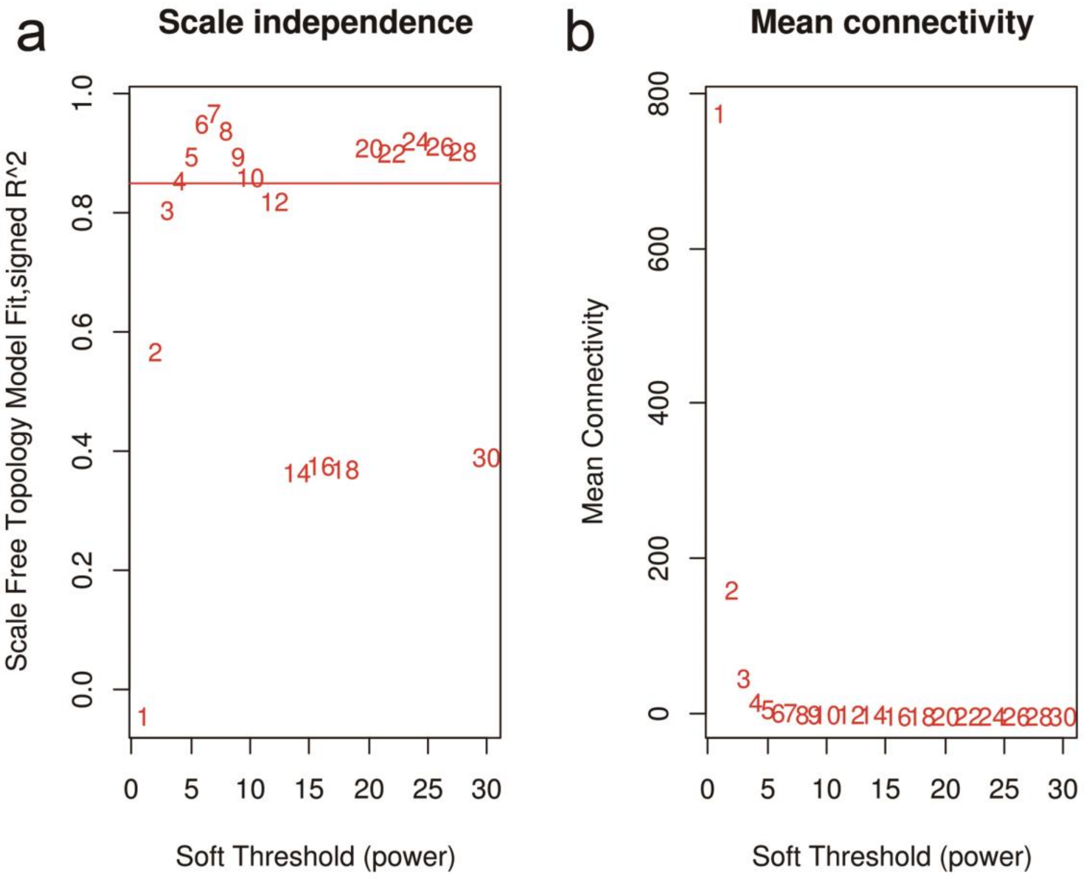

Supplementary Fig S3 Identification of key modules associated with nodule type of TNs.

(a) Dendrogram of all differentially expressed genes clustered based on a dissimilarity measure (1-TOM). The different color row below the dendrogram represents module membership clustered by the dynamic tree cut method (b) Heatmap of the correlation between module eigengenes and the clinical traits of TNs. Correlation coefficients and *P* values were presented in each square. (c) Distribution of average gene significance and errors in the modules associated with the nodule type of TNs. Module grey60 had the highest association with nodule type of TNs.

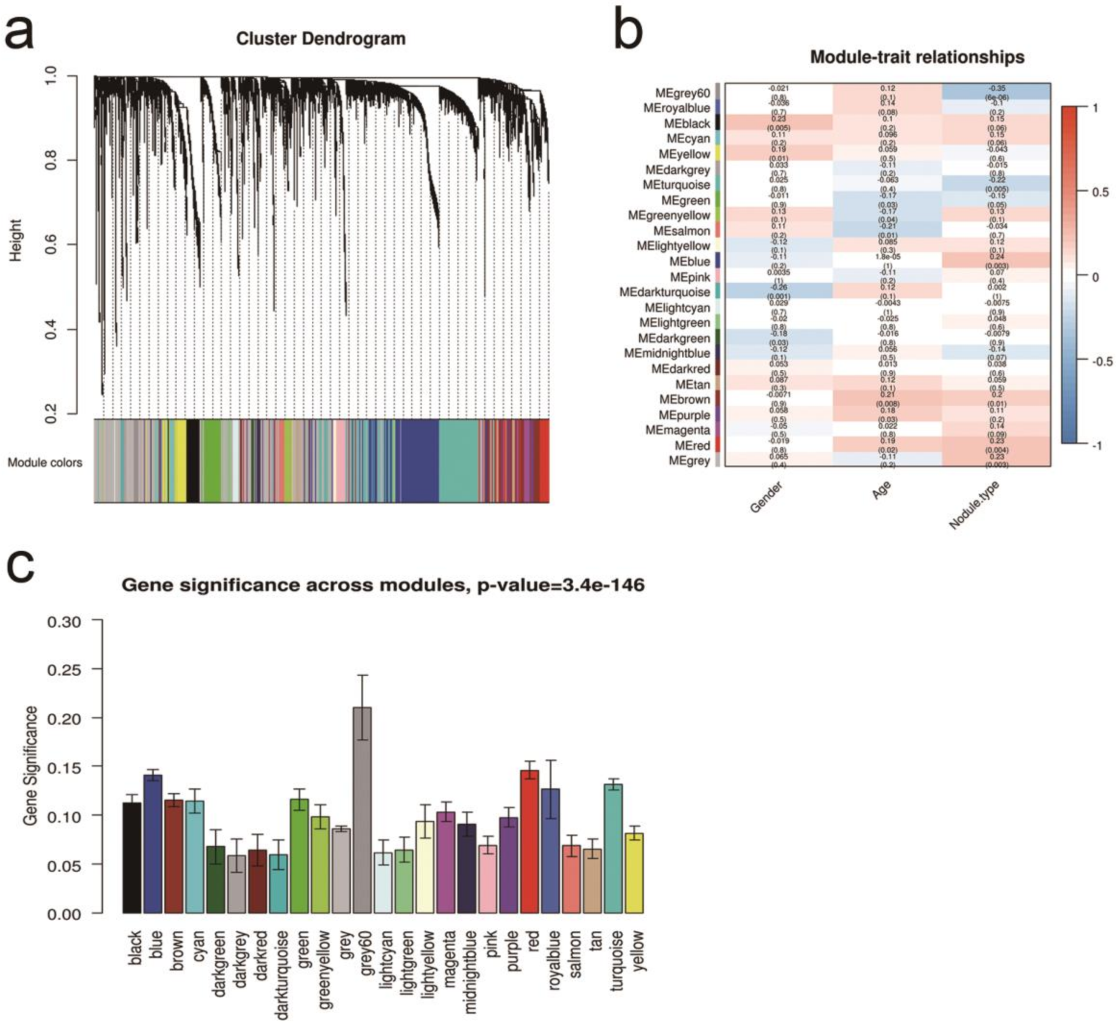

Supplementary Fig S4 Correlations between hub genes

(a) Correlation between HBB and HBA1. (b) Correlation between HBB and HERC3. (c) Correlation between HBA1 and HERC3.

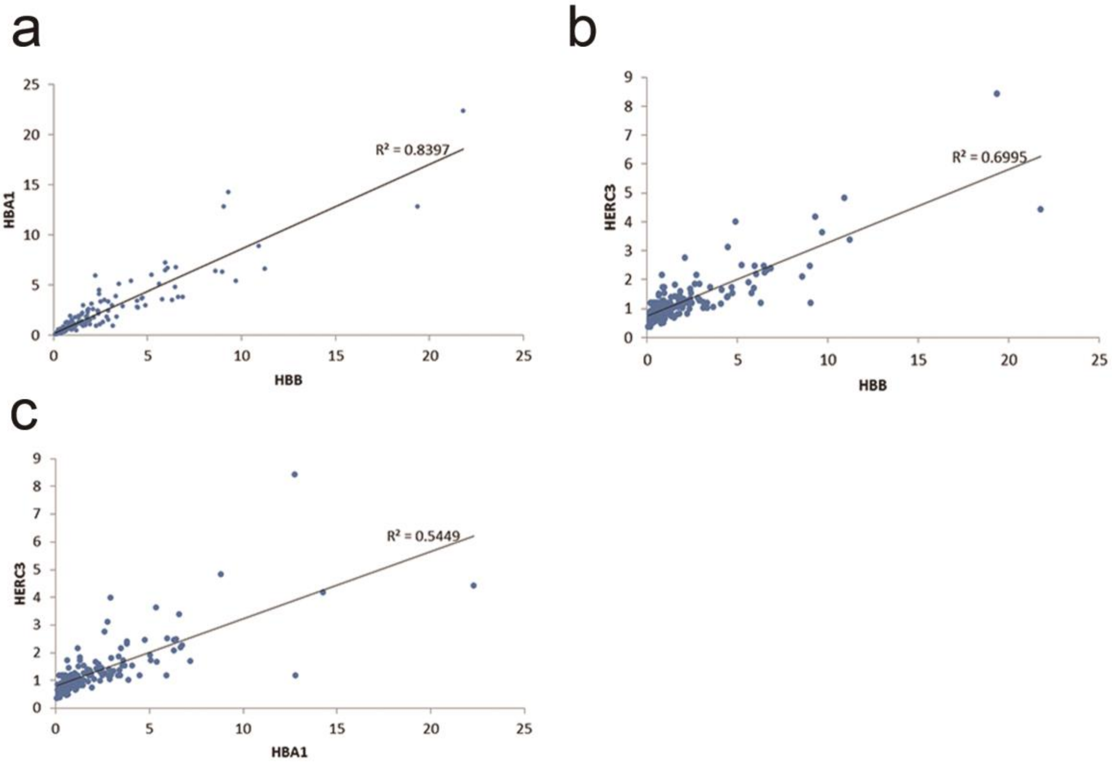

Supplement: Supplementary Materials — Supplementary Table S1: independent impact factors for thyroid nodules in the euthyroid population. Supplementary Table S2: association between inflammation and thyroid function in subjects without TNs based on propensity sore matching. Supplementary Figure S1: clustering dendrogram of 156 samples. Supplementary Figure S2: determination of soft-thresholding power (β) in WGCNA. Supplementary Figure S3: identification of key modules associated with nodule type of TNs. Supplementary Figure S4: correlations between hub genes. [file 9827349.f1.pdf]
